# Evaluations of reproductive health programs in humanitarian settings: a systematic review

**DOI:** 10.1186/1752-1505-9-S1-S1

**Published:** 2015-02-02

**Authors:** Sara E Casey

**Affiliations:** 1 Heilbrunn Department of Population and Family Health, Mailman School of Public Health, Columbia University, 60 Haven Ave, New York, NY 10032, USA

## Abstract

Provision of reproductive health (RH) services is a minimum standard of health care in humanitarian settings; however access to these services is often limited. This systematic review, one component of a global evaluation of RH in humanitarian settings, sought to explore the evidence regarding RH services provided in humanitarian settings and to determine if programs are being evaluated. In addition, the review explored which RH services receive more attention based on program evaluations and descriptive data. Peer-reviewed papers published between 2004 and 2013 were identified via the Ovid MEDLINE database, followed by a PubMed search. Papers on quantitative evaluations of RH programs, including experimental and non-experimental designs that reported outcome data, implemented in conflict and natural disaster settings, were included. Of 5,669 papers identified in the initial search, 36 papers describing 30 programs met inclusion criteria. Twenty-five papers described programs in sub-Saharan Africa, six in Asia, two in Haiti and three reported data from multiple countries. Some RH technical areas were better represented than others: seven papers reported on maternal and newborn health (including two that also covered family planning), six on family planning, three on sexual violence, 20 on HIV and other sexually transmitted infections and two on general RH topics. In comparison to the program evaluation papers identified, three times as many papers were found that reported RH descriptive or prevalence data in humanitarian settings. While data demonstrating the magnitude of the problem are crucial and were previously lacking, the need for RH services and for evaluations to measure their effectiveness is clear. Program evaluation and implementation science should be incorporated into more programs to determine the best ways to serve the RH needs of people affected by conflict or natural disaster. Standard program design should include rigorous program evaluation, and the results must be shared. The papers demonstrated both that RH programs can be implemented in these challenging settings, and that women and men will use RH services when they are of reasonable quality.

## Introduction

Increased attention to the reproductive health (RH) needs of people affected by armed conflict or natural disaster began in the mid-1990s with a few key events. The *Lancet* published an editorial identifying family planning as a complete gap in services for refugees [1]. The groundbreaking report *Refugee Women and Reproductive Health Care: Reassessing Priorities* highlighted how the health of refugee women fleeing war was further threatened by near absence of reproductive health services [2]. The 1994 International Conference on Population and Development in Cairo specifically recognized the rights of displaced populations to RH [3]. This led to the formation in 1995 of the Inter-Agency Working Group on RH in Crisis (IAWG), a consortium of non-governmental organizations (NGO), donors and United Nations (UN) agencies, to advance RH services in humanitarian settings. In 1999, the IAWG developed the *Inter-Agency field manual on reproductive health in humanitarian settings* to provide technical and program guidance to field staff [4].

In 2004, the IAWG completed a global evaluation of RH in humanitarian settings at field, agency and global levels. The evaluation found that more RH services were available than a decade earlier, although major gaps remained in most of the technical areas, with gender-based violence as the least developed technical area. Although RH services were somewhat more available for refugees living in camps, they were largely absent for internally displaced (IDP) and non-camp populations [5]. Adolescents were underserved, and safe abortion was not even assessed. The global evaluation identified a need to improve RH data collection to ensure that useful data were collected and properly interpreted, as well as for more rigorous program evaluations.

From 2012-2014, another ten years on, the IAWG conducted a second global evaluation of RH in humanitarian settings. This systematic review, one component of the 2014 global review, sought to explore the evidence regarding RH services provided in humanitarian settings. Are RH programs in these settings being evaluated? Do the programs work? What is the quality of the evaluations? Which RH services receive more programmatic and financial attention based on program evaluations and descriptive data?

## Methods

### Search strategy

This literature review summarized peer-reviewed papers published since the last global evaluation (between 2004 and 2013) that were identified via the Ovid MEDLINE database, followed by a PubMed search to pick up more recent papers not currently indexed. In addition, references for included papers were cross-checked to ensure that all relevant literature was identified and included. A combination of terms describing conflict and natural disasters were used with terms describing RH under the broad categories from the *Inter-agency field manual on reproductive health in humanitarian settings* of maternal and newborn health, family planning (FP), gender-based violence (GBV), HIV/AIDS and other sexually transmitted infections (STIs), safe abortion and adolescent reproductive health. Searches were limited to papers published in English. This initial search was broad and intended to capture all papers on RH in humanitarian settings. Papers on quantitative evaluations of RH programs, including experimental and non-experimental designs that reported outcome data were included. Descriptive quantitative studies with no specific health intervention identified and no outcomes or outputs reported (e.g., studies that reported only descriptive or baseline data) as well as purely qualitative papers were excluded. Studies were not excluded on the basis of their quality. Other inclusion and exclusion criteria are detailed in Table 1. Papers excluded under these criteria but that reported descriptive or prevalence data were logged to permit comparison of the sectoral spread of evaluation papers (the focus here) and broader prevalence or descriptive papers.

**Table 1:** Inclusion/exclusion criteria

	Included	Excluded
Topic	Papers that described RH programs to address maternal and newborn health, FP, HIV and other STIs and/or GBV (sexual violence including rape, sexual abuse and sexual exploitation, and intimate partner violence)	Papers that reported on other reproductive health topics (e.g., female genital mutilation, forced or early marriage, reproductive cancers)

Types of Papers/ Data	Quantitative evaluations of RH programs or services, including experimental and non-experimental designs that report outcome data	Descriptive quantitative papers with no specific health intervention and no outcomes (e.g., reporting only descriptive or baseline data); purely qualitative papers

Settings	Humanitarian crises in conflict, post-conflict or natural disaster settings in lower or middle income countries	Papers in locations that were not affected by armed conflict or natural disaster; that were more than ten years post-conflict; disaster settings in higher income countries

Types of publications	Papers in peer-reviewed journals	Letters, editorials, commentaries; grey literature; review papers (although these were screened for references)

Language	English	Study titles and abstracts in languages other than English

Publication date	January 2004 – December 2013	Papers published before 2004 or after 2013

### Quality assessment of the papers

The quality of each included study was assessed using criteria from the STROBE checklist for observational studies or the CONSORT checklist for clinical trials [6,7]. Papers were assigned a rating of high, medium or low quality based on the number of met criteria in a list adapted from these checklists.

## Results

**Figure 1:**
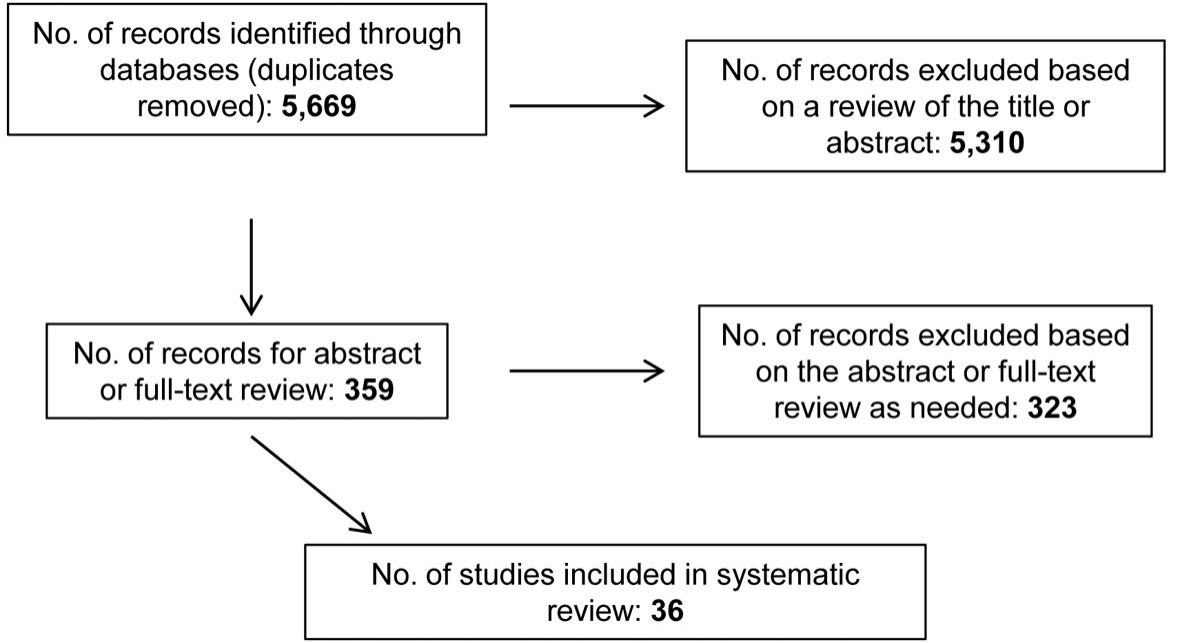
Systematic review flow chart

The search strategy yielded 5,669 papers after duplicates were removed; 5,310 were excluded based on a review of the title. Of the 359 papers for which abstract or full-text review was conducted, 323 papers were excluded, leaving 36 papers describing 30 programs (Figure 1). Of the 36 papers, 25 described programs in sub-Saharan Africa, six in Asia, two in Haiti and three reported data from multiple countries and continents. Some RH technical areas were better represented than others: seven papers reported on maternal and newborn health (including two that also covered FP), six on FP, three on GBV, 20 on HIV and other STIs and two on general RH topics (Table 2). None of the papers described safe abortion or post-abortion care programs, and five of the papers described HIV prevention programs targeting adolescents. Only six papers were classified as high quality while the majority was classified as medium quality or low quality. Fewer than half (16) of the papers reported comparison data, either in the form of pre- and post-intervention measures or intervention and comparison groups. Table 3 provides a summary of the included papers.

**Table 2:** Number of papers by RH technical component

	Number (%) of program evaluation papers (n=36*)	Number (%) of descriptive papers for comparison (n=93*)
Maternal and newborn health	7 (19%)	20 (22%)

Family planning	6 (17%)	4 (4%)

Gender-based violence	3 (8%)	32 (34%)

HIV and other STIs	20 (56%)	27 (29%)

Adolescent RH	5 (14%)	5 (5%)

General RH	2 (6%)	7 (8%)

*The sum of the RH components is greater than the total number of papers as some papers reported on multiple components.

**Table 3:** Description of papers included in the review

Author (Year)	Country	Intervention	Evaluation design	Key findings	Quality
**Maternal and newborn health**					

Ayoya et al (2013) [14]	Haiti	Established baby tents in five cities to promote and sustain optimal infant feeding practices: breastfeeding and nutrition support, infant growth monitoring, assessment of nutritional status of mother-infant pairs and pregnant women	Program data review (February 2010-June 2012) from nutritional cluster database (n=193 baby tents)	70% of infants less than 6 months old were exclusively breastfed. 10% of “mixed feeders” less than 6 months changed to exclusive breastfeeding while enrolled.	Low

Howard et al (2011)^1^[12]	Guinea	Seconded refugee health workers to health facilities serving refugees, provided free RH services and trained refugee women as lay health workers	Cross-sectional post-intervention multi-stage cluster survey in intervention area of women (n=444) and men (n=445) of reproductive age (Liberian and Sierra Leonean refugees) living in one of 48 refugee camps in Guinea in 1999.	Higher odds of facility delivery for those exposed to intervention education activities (OR=2.03, 95%CI 1.23-3.01), formally educated (OR=1.93, 95%CI 1.05-3.92), or grand multipara (OR= 2.13, 95%CI 1.21-3.75). No significant differences found in maternal health knowledge or attitudes.	Medium

Krause et al (2006) [9]	Global (9 countries)	Improved availability of basic and comprehensive EmONC services in 12 conflict affected settings in 9 countries, Jan 2001-Apr 2005	Pre and post intervention facility assessments (n=31 health facilities)	Increased availability of EmONC 24 hours a day. CEmONC facilities increased from 3 facilities at baseline to 10 at endline; BEmONC facilities increased from 2 at baseline to 10 at endline. The number of signal functions available increased in all 31 facilities.	Medium

Lori et al (2010) [15]	Liberia	Trained traditional midwives in maternal care using the home-based life-saving skills series in 2006	Pre- & immediate post-training assessments (n=412 traditional midwives), 1-year follow up assessment (n=389)	Mean scores in 4 topic areas: 1) first actions, 2) post-partum hemorrhage 3) woman referral, 4) baby referral, improved from pre- to post-test and remained stable one year later (p<.001 for all 4 topics)	Medium

Purdin et al (2009) [8]	Pakistan	Established EmONC facilities, trained Afghan refugee community members on safe motherhood, linked primary health care with education on danger signs of pregnancy and the importance of skilled birth attendance, and improved the health information system.	Program data review 2000-2007	Maternal mortality ratio improved from 291 per 100,000 live births in 2000 to 102 in 2004. Case fatality rate for obstetric complications=0.2%. Skilled birth attendance increased from 5% in 1996 to 67% in 2007. Complete ANC coverage increased from 49% in 2000 to 90% in 2006; post-natal coverage increased from 27% in 2000 to 85% in 2006.	Medium

**Maternal and newborn health and family planning**					

Mullany et al (2010) [11]	Burma	Trained community-based skilled health workers in basic EmONC, evidence-based ANC and FP in Shan, Mon, Karen, and Karenni regions of Burma	Pre (2006) & post (2008) intervention cross-sectional two-stage cluster surveys in intervention areas of ever married women of reproductive age: n=2,889 at baseline, n=2,442 at endline	Use of modern FP methods increased from 24% to 45% (PRR 1.88, 95%CI 1.63-2.17). Unmet need for FP decreased 35% (95%CI 28%-40%). Skilled birth attendance increased from 5% to 49% (PRR=9.55, 95%CI 7.21-12.64).	High

Viswanathan et al (2012) [13]	Afghanistan	Deployed CHWs to promote use of RH services in community and at health facilities	Data derived from the Afghanistan Health Survey 2006: multistage cluster survey in 29 provinces (n=8,281 women)	Presence of female CHW in community is associated with increased use of FP (OR=1.61, 95%CI 1.21-2.15), ANC (OR=2.71, 95%CI 1.87-3.92) and skilled birth attendant at last delivery (OR=1.75, 95%CI 1.18-2.58). These associations were not significant with a male CHW.	Medium

**Family planning**					

Casey et al (2013) [16]	Northern Uganda	Provided short-acting, long-acting and permanent FP methods via mobile outreach teams and strengthened public health center provision of short and long acting FP methods	Baseline (2007) and post-intervention (2010) cross-sectional multi-stage cluster surveys in intervention area of women of reproductive age: n=905 at baseline, n=873 at endline	Current use modern FP methods increased from 7.1% to 22.6% (OR=3.34, 95%CI 2.27-4.92); use of LAPM increased 1.2% to 9.8% (OR=9.45, 95%CI 3.99-22.4). Unmet need for FP decreased from 52.1% to 35.7% (OR=0.47, 95% CI 0.37-0.60).	High

Howard et al (2008)^1^[17]	Guinea	Refugee health workers seconded to health facilities provided free RH services and trained refugee women as lay health workers	Cross-sectional post-intervention multi-stage cluster survey in intervention area of women (n=444) and men (n=445) of reproductive age (Liberian and Sierra Leonean refugees) living in one of 48 refugee camps in Guinea in 1999.	Approval of FP was high, but more than 40% had not discussed FP with partner. Current use of modern FP (17%) was higher than in country of origin (3.9%) or host country (4.1%). Perceived service quality was most important determinant in choice of where to get FP.	Medium

Huber et al (2010) [18]	Afghanistan	Improved access to FP using CHWs and community-based distribution of short acting methods	Baseline (2004) and endline (2006) cross-sectional surveys using lot quality assurance sampling; verification of FP use via home visits of 150 FP users per CHW	Current FP use increased by 24-27%, with injectables contributing most to the increase.	Low

Raheel et al (2012) [19]	Pakistan	Provided subsidized or unsubsidized health care to Afghan refugees in Karachi	Cross-sectional study in 2008 using systematic random sampling of 2 comparison groups: married Afghan women of reproductive age receiving subsidized care (n=325) and unsubsidized care (n=325)	Refugee women receiving subsidized care were more likely to have heard of FP (OR=10.12 95%CI 6.7-15.31) and currently use FP (OR=3.65, 95%CI 2.61-5.10).	High

**Gender-based violence**					

Bass et al (2013) [20]	Democratic Republic of the Congo (DRC)	Adapted group cognitive processing therapy (1 individual session and 11 group sessions) provided by paraprofessionals supervised by psychosocial staff and clinical experts	Random assignment of 16 villages to intervention group (8) or individual support (8) for female sexual violence survivors in 2011	65% in intervention group and 52% in control group completed all 3 measures. Improvements in all 3 sets of symptoms were significantly greater in therapy group than in individual support group. Mean scores for combined depression and anxiety improved significantly more in the therapy group compared to the individual support group (p<0.001 for all comparisons).	High

Hustache et al (2009) [21]	Republic of Congo	Provided medical care and psychological support to women raped by an unknown perpetrator in military clothing	Initial assessment January 2002-April 2003 (n=159 female survivors of rape); follow-up 1-2 years post-treatment, June-July 2004 (n=70)	56 women were evaluated using the Global Assessment of Functioning (GAF) scale at both time periods, and global functioning significantly improved (p=.04); this improvement was maintained 1-2 years later	Medium

Smith et al (2013) [22]	DRC, Ethiopia, Kenya, Jordan	Multimedia training tool for health providers to encourage competent, compassionate, and confidential clinical care for rape survivors	Assessment pre-training and 3 months after, medical record review, in-depth interviews (November 2010 to June 2012)	Although negative attitudes did not significantly decrease, respect for patient rights increased (p<.05), and provider practice improved from before the training to 3 months post-training (p<.01).	Medium

**HIV/AIDS and other STIs**					

Ahoua et al (2010) [24]	Northern Uganda	PMTCT program including either short-course AZT or single dose nevirapine and follow-up for 18 months post-partum including infant HIV testing	Retrospective record review of all mother-infant pairs enrolled July 2000-July 2005 (n=517). Cross-sectional survey of infant status at 18 months following tracing of mother-infant pairs who were lost to follow up (n=327 women and 368 babies).	53% of mother-infant pairs were lost to follow-up before completing infant testing at 18 months; the risk of death or being lost to follow-up was higher among infants with no or incomplete intrapartum ARVs (OR=1.9, 95%CI 1.07–3.36) and of weaning before age 6 months (OR=2.55, 95%CI 1.42–4.58).	Medium

Atwood et al (2012)^3^[35]	Liberia	Evidence-based HIV prevention curriculum adapted for in-school Liberian youth. The 8-modules promoted positive condom attitudes and increased skills and self-efficacy to refuse sex, negotiate condom use and use condoms effectively.	Attention-matched, group RCT: 4 matched pairs of schools randomly assigned to HIV prevention curriculum or general health curriculum. Students completed baseline, immediate post-test, 3- and 9- month follow-up surveys to assess program efficacy (n=740 completed all measures)	The intervention significantly improved protective peer norms (p<.05) and positive condom attitudes (p<.05) at the 9 month follow-up. Among those who were sexually active at baseline, the intervention group used condoms more consistently in the last 3 months (p<.05) at the 9-month follow-up. The intervention did not impact sexual initiation or multiple sex partnerships.	Medium

Atwood et al (2012)^3^[36]	Liberia	Evidence-based HIV prevention curriculum adapted for in-school Liberian youth. The 8-modules promoted positive condom attitudes and increased skills and self-efficacy to refuse sex, negotiate condom use and use condoms effectively.	Attention-matched, group RCT: 4 matched pairs of schools randomly assigned to HIV prevention curriculum or general health curriculum. Students completed baseline, immediate post-test, 3- and 9- month follow-up surveys to assess program efficacy (n=714 who responded to questions about transactional sex)	Risk behaviors for adolescents who engaged in transactional sex were no different in the intervention or control groups.	Medium

Bannink-Mbazzi et al (2013) [23]	Northern Uganda	PMTCT program including couple VCT, care and treatment for HIV+ individuals, home-based care, partner involvement, follow-up at 18 months post-partum including infant HIV testing	Retrospective record review of PMTCT program data 2002-2011	Of 140,658 women starting ANC, 94.4% received HIV testing. Testing of male partners increased from 5.9% in 2002 to 75.8% in 2011 (p=.001) compared to 15.5% nationally. 79% of HIV+ women started ARVs, compared to 52% nationally. HIV prevalence among exposed infants tested by 18 months decreased from 10.3% in 2004 to 5.0% in 2011 (p=.001).	Medium

Casey et al (2006)^2^[37]	Sierra Leone	HIV/AIDS and STI prevention program comprised of intensive outreach education by peers including a focus on improving negotiation skills and distribution of free condoms targeting youth	Baseline (2001) and post intervention (2003) cross-sectional surveys using purposive quota sampling of youth: n=244 female, 293 male (baseline); n=250 female, 299 male (endline)	Respondents able to name 3 effective means of avoiding AIDS increased from 4% to 36% among female youth and from 4% to 45% among male youth; reported condom use at last sex increased from 16% to 46% (female) and from 16% to 37% (male) (p<.01 for all comparisons).	Medium

Chen et al (2008)^1^[39]	Guinea	Refugee health workers seconded to health facilities provided free RH services and trained refugee women as lay health workers	Cross-sectional post-intervention multi-stage cluster survey in intervention area of women (n=444) and men (n=445) of reproductive age (Liberian and Sierra Leonean refugees) living in one of 48 refugee camps in Guinea in 1999.	Self-reported STI symptoms were common: 30% among women and 24% among men. Only 25% correctly named key STI symptoms. Respondents citing program facilitators as sources of information were more likely to correctly name key STI symptoms (OR=5.2, 95% CI 1.9-13.9 (men)) and identify effective means of protecting against STIs (OR=2.9, 95% CI 1.5-5.8 (men)) and (OR=4.6, 95%CI 1.6-13.2 (women)).	Medium

Ciccio and Sera (2010) [41]	Northern Uganda	HIV/AIDS prevention activities with youth including media campaigns, peer counseling, life skills training, and activities for youth in particularly vulnerable circumstances to spread prevention messages and help them develop the skills necessary to protect themselves.	Cross-sectional post-intervention survey using lot quality assurance sampling in intervention area (n=1,781 youth age 15-24) in 2008	29% had comprehensive HIV prevention knowledge (knew 3 main means of prevention and rejected common misconceptions). 86% knew where to get tested for, but only 51% had been tested and received their result in the last 12 months. Gender, geographical location, marital status and education were associated with this knowledge (p<.001)	Medium

Culbert et al (2007) [33]	DRC	Voluntary counseling and HIV testing (VCT), care and treatment for HIV+ individuals, HIV prevention activities	Program data review: May 2002-Jan 2006	11,076 people received VCT, of whom 19% were HIV+; 94% of these received follow-up care in the HIV clinics. 12-month mortality among ART patients was 7.9% (95%CI 3.6-12.1), and 12-month loss to follow-up was 5.4% (95%CI 3.2-7.5), both comparable to stable low resource settings. Only 5 of 66 ART patients experienced treatment interruption during violent period of May-June 2004.	Medium

Garang et al (2009) [26]	Northern Uganda	Care and treatment for HIV+ individuals	Cross-sectional study using systematic sampling of self-reported adherence over 4-day period in February 2008 (n=200 adults on ART)	Mean 4-day adherence (self-reported) was 99.5%, with no difference between IDPs and non-IDPs. Being on a 1st line ART regimen (OR=22.2, 95%CI 1.5-333.3), feeling facility staff were condemning (OR=22.2, 95%CI 1.5-333.3), and lack of privacy at facility (OR=9.7, 95%CI 0.9-111.1) were associated with non-adherence.	High

Kiboneka et al (2008)^4^[28]	Northern Uganda	Care and treatment for HIV+ individuals, facility and home-based care, mobile clinics to IDP camps	Prospective cohort study using program data June 2005 - Feb 2008 (n=57 HIV+ children receiving combination ART)	Adherence was consistently excellent in 92% of patients. No deaths and no major opportunistic infections were recorded after initiation of ART.	Medium

Kiboneka et al (2009)^4^[27]	Northern Uganda	Care and treatment for HIV+ individuals, facility and home-based care, mobile clinics to IDP camps	Prospective cohort study using program data, June 2005 - Jan 2008, (n=1,625 HIV+ adults receiving combination ART)	The mortality incidence rate was 3.48 (95%CI 2.7-4.3) per 100 person years. Of patients with adherence data, 92% had adherence greater than 95%. 4.3% of patients died during follow-up, a mortality rate comparable to ART patients in stable settings. Lower mortality was associated with female sex, higher baseline CD4 count and ≥95% adherence. IDP camp residence and age were not associated with mortality outcomes.	Medium

Larsen et al (2004)^2^[38]	Sierra Leone	HIV/AIDS and STI prevention program comprised of intensive outreach education by peers including a focus on improving negotiation skills and distribution of free condoms targeting commercial sex workers (CSW) and military men	Baseline (2001) and post intervention (2003) cross-sectional surveys using purposive quota sampling: n=201 sex workers, 202 military men (baseline); n=202 sex workers, 205 military men (endline)	Those able to name 3 effective means of avoiding AIDS increased from 5% to 70% among CSWs and from 11% to 75% among military men. Reported condom use at last sex increased from 38% to 68% (CSW) and from 39% to 68% (military) (p<.01 for all). Although the proportions of both CSWs and military men who believe HIV+ people should be treated or counselled increased, the proportions believing they should be isolated or reported did not change.	Medium

O'Brien et al (2010) [29]	Global	Programs of care and treatment for HIV+ individuals in conflict and post-conflict settings	Program data review 2005-2009 (n=20 programs with complete data and n=4,145 HIV+ adults on ART with complete data)	64% of ART patients remained on ART, 10% died, 11% were lost to follow-up. Median 12-month mortality and loss to follow-up were 9% (95%CI 8.8-9.1) and 11% (95%CI 9-12) respectively. Median 6-month CD4 gain was 129 cells/mm^3^.	Medium

Pyne-Mercier et al (2011) [30]	Kenya	Care and treatment for HIV+ individuals	Retrospective record review for clients on ART during post-election violence, Dec 30, 2007 - Feb 28, 2008, and same time period 1 year earlier (n=2,534 HIV+ adults)	The odds of treatment interruption were 71% (95%CI 34-118) higher during the post-election violence period compared to 1 year earlier. Men (OR=1.4, 95%CI 1.1-1.8) and those traveling ≥3 hours to clinic (OR=1.9, 95%CI 1.3-2.7) were more likely to experience treatment interruption.	High

Rutta et al (2008) [25]	Tanzania	2-year pilot PMTCT program in refugee camp: community education, training providers, VCT, infant feeding, counseling, administration of nevirapine	Program data review Oct 2002 - June 2004 (n=6 health facilities)	92% of ANC clients were tested for HIV. 93% of HIV+ women agreed to take nevirapine at 34 weeks of gestation. 36% of the HIV+ women were repatriated before delivery, but 98% of those remaining took nevirapine at the start of labor and their infants received nevirapine within 72 hours. Only 15% of HIV-exposed infants were tested at 18 months due to repatriation, death or refusal of testing.	Medium

Tanaka et al (2008) [42]	Tanzania	HIV/AIDS prevention including youth peer education, VCT, free condom distribution in Nyarugusu refugee camp	Post-intervention survey of systematically selected Congolese refugees of reproductive age (n=570 male and 570 female) living in the refugee camp in 2005	HIV risk increased after displacement due to increased transactional sex and forced sex (p<.001). Condom use at last sex with a non-regular partner was 14% and associated with citing the program health teams as a leading source of influence regarding HIV prevention	Medium

Vreeman et al (2009)^5^[31]	Kenya	Care and treatment for HIV-infected children	Retrospective cohort analysis of HIV+ children under 14 years seen from Oct-Dec 2007 in 18 clinics (n=2,585), and then followed from Dec 2007 until April 2008.	93% of HIV-infected children returned to care in the 4 months after the violence, and 98% of children on ART reported perfect adherence during last 7 days (p<.001). Children on ART were more likely to return than those not on ART (OR=1.4, 95%CI 1.2-1.6). Orphan status and sex were not associated with return to clinic.	Medium

Walldorf et al (2012) [34]	Haiti	HIV/AIDS clinical services including VCT, PMTCT, care and treatment for HIV+ individuals	Program data Oct 2008-May 2010 comparing pre-earthquake (prior to Dec 2009) to post-earthquake outcomes (n=126 facilities)	Mean monthly enrollment for VCT, PMTCT and ART services were from 41-46% of baseline levels in Jan 2010 but rose to 79-89% of baseline levels in May 2010. Current ART patients rose 3.6% Jan – May 2010 compared to a 9.8% increase during the same period in 2009.	Medium

Woodward et al (2011)^1^[40]	Guinea	Refugee health workers seconded to health facilities provided free RH services and trained refugee women as lay health workers	Cross-sectional post-intervention multi-stage cluster survey in intervention area of women (n=444) and men (n=445) of reproductive age (Liberian and Sierra Leonean refugees) living in one of 48 refugee camps in Guinea in 1999.	HIV knowledge was high. Participants exposed to program peer education had higher odds of reporting changes in sexual behavior to avoid HIV (OR=2.5, 95%CI 1.5-4.1). Exposed participants were less likely to report staying faithful (OR=0.6, 95%CI 0.4-0.9) and more likely to report fewer sex partners (OR=1.7, 95%CI 1.05-2.85).	Medium

Yoder et al (2012)^5^[32]	Kenya	HIV/AIDS care and treatment for HIV-infected children	Retrospective cohort analysis for 3 time periods: pre-election, Oct 26-Dec 25 2007; immediately post-election, Dec 26, 2007 - Apr 15, 2008; and long-term post-election, Apr 16-Dec 31, 2008 (n=2,549 HIV+ children)	Children on ART had less initial loss to follow-up (p<.01) and less complete loss to follow-up (p<.0001) than children not on ART. Immediately post-election, 8.2% of children on ART had imperfect medication adherence, and 9.0% long-term post-election.	Medium

**General RH**					

McGinn & Allen (2006) [44]	Guinea	Literacy training using RH information as the content and participatory adult education techniques for Sierra Leonean and Liberian women living in refugee camps	Post-intervention cross-sectional survey of RH literacy program students who participated in 1999, 2000 and 2001 RH literacy courses and were still in the area in 2002 (n=549)	The proportion of women who reported communication with their partners on RH topics increased to 87% (p<.001). Current use of FP was 50%. The proportion of women who reported feeling more empowered than other women increased from 32% (based on recall) to 82% after the program (p<.001).	Medium

Sullivan et al (2004) [43]	Thai-Burma border	Program to improve quality of RH services and build health providers' capacity in monitoring and evaluation	Pre- and post-intervention facility audits, observations of client-provider interactions during ANC and FP visits, client exit interviews (2001-2003)	Improved program readiness contributed to improved quality of information given to clients, technical competence and integration of services, although some contradictory findings from client exit interviews warrant further exploration.	Low

^1,2,3,4,5^These articles refer to the same program.

Of the 323 papers reviewed and excluded, 93 papers reported descriptive or prevalence data on RH in crisis settings. Again, some RH technical areas were better represented than others: 20 papers on maternal and newborn health (including one that also reported on FP and one that also looked at GBV), four on FP, 32 on GBV, 27 on HIV or other STIs (only six of which mentioned other STIs), seven papers on general RH and five on adolescent RH (specifically HIV, GBV or FP) (Table 2).

### Maternal and newborn health

Seven of the 36 papers described evaluations of maternal and newborn health programs, including two programs that also addressed family planning. The papers covered a range of topics including emergency obstetric and newborn care (EmONC), antenatal care (ANC) and the training of traditional birth attendants or community health workers (CHWs) to improve maternal health outcomes.

Two papers described the outcomes of programs to improve EmONC services, the first for Afghan refugees in Pakistan [8] and the second in humanitarian settings in nine countries [9]. Although not all supported facilities met the WHO criteria of fully functional EmONC facilities [10], the papers reported greater availability post-intervention of EmONC services 24 hours a day and subsequent increased use of those services in most facilities. The authors of both papers described challenges in calculating the UN process indicators for EmONC^a^ at baseline [10], primarily due to the absence of key data from delivery registers; however, both reported these indicators at endline.

Other program approaches to improve maternal and newborn health involved training mobile health workers to provide elements of basic EmONC plus blood transfusion and ANC in eastern Burma [11]; seconding refugee health workers to health facilities serving the refugee population and training refugee women to promote RH in the community in Guinea [12]; and training CHWs in Afghanistan to strengthen the link between the community and formal health services [13]. All three papers reported increased use of skilled birth attendants post-intervention. The Afghanistan study, however, found that only the presence of a female CHW was associated with increased skilled birth attendance; the association was absent with male CHWs. One paper assessing the effectiveness of baby tents (clean spaces to support mothers to practice healthy infant feeding) established in Haiti found that 70% of babies less than six months old were exclusively breastfed and 10% of non-exclusively breastfed infants moved to exclusive breastfeeding while enrolled [14]. Finally, an evaluation of a home-based lifesaving skills training for traditional midwives in Liberia found that midwives’ knowledge improved from pre to post training and remained stable one year later [15].

### Family planning (FP)

Six papers described FP programs, including two that also described maternal and newborn health outcomes. Programs used different strategies to improve FP use: providing the full range of FP methods, including long-acting and permanent methods, via mobile clinics and strengthening health centers’ provision of short- and long-acting FP in northern Uganda [16]; training mobile health workers to provide short-acting methods in eastern Burma [11]; seconding refugee providers to health facilities serving refugees to provide FP and training female CHWs to promote FP use in Guinea [17]; and training CHWs to conduct FP education and provide short acting methods in Afghanistan [18]. All four papers reported that contraceptive prevalence increased from baseline or was higher than national levels. Additional papers found that the presence of a female CHW was associated with higher FP use in Afghanistan [13], and that contraceptive use was higher among Afghan refugee women in Pakistan who received subsidized health services than among those with access to un-subsidized services [19].

### Gender-based violence (GBV)

Although the literature search included broader terms related to GBV, all three included papers focused specifically on care for survivors of rape. Two papers reviewed the effectiveness of psychosocial interventions for survivors. A randomized controlled trial in the Democratic Republic of the Congo (DRC) on the effectiveness of group cognitive processing therapy versus individual support to female survivors of rape found that those who received group psychotherapy showed greater improvement in depression, anxiety and post-traumatic stress disorder (PTSD) symptoms six months after treatment compared to those in the control group [20]. The second paper found that the global functioning of survivors in the Republic of Congo improved following post-rape psychological care, and improvement was maintained one to two years later although high loss to follow up weakened these results [21]. The third paper reviewed the effects of a multi-media training tool for clinical care for rape survivors on the knowledge, attitudes and practices of health providers in four conflict settings [22]. The authors found that although negative attitudes towards survivors did not significantly change, respect for patient rights increased and provider practice improved from pre-training to three months post-training.

### HIV and other sexually transmitted infections (STIs)

More papers (20) focused on HIV and other STIs than any other RH component; however only three of these reported on STIs other than HIV. Three papers reported results of retrospective record reviews to evaluate programs to prevent mother to child transmission of HIV (PMTCT), two in northern Uganda and one in a refugee camp in Tanzania. One program found that higher proportions of HIV-positive pregnant women identified in ANC used anti-retroviral prophylaxis in northern Uganda compared with the national average [23]. The other two programs reported high numbers lost to follow-up before completing infant HIV testing at 18 months. In one study, this was primarily due to a lack of understanding of its importance and infant death; incomplete or no ARV prophylaxis, early weaning and prolonged breastfeeding were associated with increased risk of loss to follow-up and infant death [24]. In the final study, more than two-thirds of the HIV-infected women were repatriated to their home country before delivery; among those who delivered in the camp, nevirapine uptake was 98% [25].

Eight papers reported the outcomes of anti-retroviral therapy (ART) programs for HIV-positive adults or children in East Africa, Haiti and globally. Three papers found that ART patients in northern Uganda had mortality rates and adherence comparable to or better than ART patients in stable settings or who were not displaced [26-28]. Similarly, a review of the data from 24 ART programs in conflict or post-conflict settings found that patient outcomes were comparable to those in stable settings [29]. Five papers examined the effect of a crisis on ART programs: the post-election violence in Kenya in early 2008 [30-32], acute conflict in DRC in 2004 [33] and the earthquake in Haiti in 2010 [34]. Notably, although the papers found higher rates of treatment interruption immediately post-disaster, generally services were quickly re-established and patient attendance and adherence rebounded soon after.

Eight papers reported HIV and/or STI knowledge, attitudes and behavior results following HIV prevention programs. Two papers reported on a group randomized controlled trial to evaluate the impact of an evidence-based HIV prevention intervention on sexual risk behaviors of in-school 6^th^ graders in Liberia [35,36], and six used post-intervention surveys to assess program effectiveness in four African countries [37-42]. All of the papers reported mixed results of their prevention programs regarding some elements of knowledge and behavior change; however, the four that follow reported more positive results. A comparison of pre- and post-intervention survey data in Sierra Leone found that HIV-related knowledge and condom use increased among adolescents [37], commercial sex workers and military personnel [38] following an HIV prevention program including intensive IEC activities and distribution of free condoms. Two papers on refugee camps in Guinea reported that exposure to program peer educators was associated with improved HIV and STI knowledge and changed behavior to prevent HIV [39,40].

## General RH

Two papers reported on unique efforts related to reproductive health. A program to improve and measure the quality of RH services at a clinic serving Burmese refugees and migrant workers on the Thailand-Burma border improved the quality of care, and also increased staff skills and motivation to collect and use data to make program decisions [43]. An evaluation of a literacy program that used RH content in Guinea found that refugee women who completed the program reported high knowledge on maternal and newborn health, HIV and STIs; increased use of FP; and a marked increase in feelings of empowerment [44].

## Discussion

This review found that some RH programs in crisis settings have been evaluated although most evaluations were medium in quality, suggesting limitations in study design and analysis. Most of the papers reported generally positive results suggesting that these programs are likely well-designed and reasonably well-implemented. The papers demonstrated both that RH programs can be implemented in these challenging settings and that women and men will use RH services when they are of reasonable quality. In comparison to the program evaluation papers identified, three times as many papers were found that reported RH descriptive or prevalence data in humanitarian settings. While data demonstrating the magnitude of the problem are crucial and were previously lacking, the need for RH services and for evaluations to measure their effectiveness is clear [45,46]. It is critical to more directly link research to interventions and increase the evidence base for RH service delivery strategies in humanitarian settings. This includes not only the research but also publication and sharing of results. An increased focus on implementation science is needed to explore how best to improve delivery and use of RH services as well as the use of research to improve practice [47].

Although published articles are not representative of RH programs implemented in humanitarian settings as most programs do not publish their results, they may reflect relative attention, both programmatic and financial, to particular areas. A preponderance of papers reported on HIV/AIDS programs although few mentioned other STIs. While GBV was under-represented among program evaluations, one-third (32) of the descriptive papers reported prevalence and types of sexual violence perpetrated in humanitarian settings. This suggests that GBV does, in fact, receive attention in research, although perhaps less in programming which when implemented may be only rarely evaluated. FP, on the other hand, was under-represented among both program evaluations and descriptive papers suggesting that FP overall receives less attention than the other RH components. Adolescents often face additional barriers to meeting their RH needs [48], but only four HIV prevention programs targeted adolescents and no papers evaluated adolescent-friendly RH services. No papers mentioned safe abortion which remains virtually unavailable in humanitarian settings [49], nor post-abortion care.

Programs requiring long-term follow-up faced specific challenges introduced by the instability of crisis settings and associated population movements. Some of these challenges, such as brief interruptions to treatment that arose during incidents of crisis, can and should be managed or prevented with planning, as demonstrated in the response to post-election violence in Kenya [30,32] and an upswing in violence in DRC [33]. Training refugee or IDP health workers, who would likely move with their community, may be a potential strategy for ensuring continued access to care for displaced people after they return home. Additional challenges to the implementation of RH programs were identified in the papers. For example, highly trained health workers are needed to provide RH services, and they may require updated competency-based training, particularly for EmONC, long-acting and permanent FP and clinical care for survivors of rape. The evaluation of a training tool for providers suggested that although attitudes are challenging to change, care for survivors of rape can be improved [22].

Proven evidenced-based strategies should be adapted and implemented in humanitarian settings. For example, EmONC is crucial to reduce maternal morbidity and mortality, and is thus a component of the minimum standard in humanitarian RH service delivery (the Minimum Initial Service Package) [4]. Yet, only three of the seven maternal and newborn health programs that were evaluated aimed to improve the availability of these critical services. Only one of the evaluated programs improved the availability of long-acting or permanent FP methods; the other programs were generally limited to short-acting methods, despite evidence that a broad choice of methods is an essential component of good FP programming and also associated with increased use [50-52]. Although a foundation in social change theory has been shown to be important for behavior change [53], only one of the HIV prevention programs appears to have had such a base [35,36]. Behavior change communication efforts implemented in humanitarian settings should adapt such proven evidence-based strategies. Moreover, it is critical that best practices be shared across the humanitarian and development fields. While the humanitarian field has adapted strategies that have been successful in development settings for many RH components, response to sexual violence is one area where the humanitarian field may be in advance of the development field, and it is crucial that these programs be implemented, rigorously evaluated and published. Further, it would be useful for programs (and journals) to publish results of programs that were unsuccessful so others may learn from those experiences.

Fewer than half of the papers used any kind of comparison, either between pre- and post- measures or between intervention and comparison groups. This is not a call for more randomized controlled trials, however, since randomizing clients is not often appropriate, due to the fundamental principle of client choice in FP and GBV programming [54]. Evaluations using pre- and post-intervention measures or quasi-experimental designs may be appropriate, particularly where a program strategy is implemented in phases and a group that has not yet received the intervention serves as a comparison for a group in an earlier phase of the program. In addition, the challenges to collecting data in humanitarian settings are well-recognized [55,56], and population-based surveys may be particularly challenging in these unstable and insecure settings [57]. Therefore, other rigorous measures of program quality that are feasible to collect should be explored. For example, the UN process indicators of EmONC were developed to monitor interventions proven to reduce maternal mortality without the limitations and expense of a maternal mortality survey by using information available at health facilities [10,58]. What similar practical approximations could be used to measure the success of FP and GBV programs? It is plausible that evaluations of clinical HIV programs were in the majority because program quality could be measured using clinical data (patient adherence and outcomes) that were routinely collected. Challenges to collecting appropriate data have been noted [5,9]; increased effort should be put into routine data collection to ensure that good quality data to measure standard indicators are collected, and shared. This may mean adapting registers to capture data on, for example, obstetric complications or to record new, continuing and switching FP clients.

Limitations of this review include its restriction to quantitative methodologies and to papers published in English, which may have excluded relevant publications. The selected search parameters may have missed papers that did not explicitly refer to conflict or humanitarian settings or natural disasters, or the general RH topics that were searched in the title, abstract or key words. While the included papers may be representative of peer-reviewed published literature, they are not representative of RH programming in humanitarian settings: humanitarian agency staff may not have time to write up results for publication and negative or null findings may be difficult to publish.

Program evaluation and implementation science should be incorporated into programs to determine the best ways to serve the RH needs of people affected by conflict or natural disaster. Standard program design should include rigorous program evaluation [59] and improved routine data collection. The results must be shared so that proven evidence-based strategies for RH are implemented in humanitarian settings. These papers demonstrated both that RH programs can be implemented in these challenging settings, and that women and men will use RH services when they are of reasonable quality.

## List of abbreviations used

ANC: antenatal care; ART: anti-retroviral therapy; CHW: community health worker; DRC: Democratic Republic of the Congo; EmONC: emergency obstetric and newborn care; FP: family planning; GBV: gender-based violence; IAWG: Inter-Agency Working Group on Reproductive Health in Crisis; NGO: nongovernmental organization; PMTCT: prevention of mother to child transmission of HIV; PTSD: post-traumatic stress disorder; RH: Reproductive health; STIs: sexually transmitted infections; UN: United Nations.

## Competing interests

The author declares that she has no competing interests.

## Authors’ information

The author is a member of the Steering Committee of the Inter-Agency Working Group on RH in Crises.

## Endnotes

^a^The eight UN process indicators for EmONC were developed to monitor progress in the prevention of maternal and perinatal deaths:

    1. Availability of EmONC: at least 5 EmONC facilities (including at least one comprehensive facility) for every 500,000 population

    2. Geographical distribution of EmONC facilities

    3. Proportion of all births in EmONC facilities

    4. Met need for emergency obstetric care: proportion of women with major direct obstetric complications who are treated in EmONC facilities (acceptable level is 100%)

    5. Caesarean sections as a proportion of all births (acceptable level between 5 and 15%)

    6. Direct obstetric case fatality rate (acceptable level is less than 1%)

    7. Intrapartum and very early neonatal death rate

    8. Proportion of maternal deaths due to indirect causes in emergency obstetric care facilities

## Declarations

This article has been published as part of *Conflict and Health* Volume 9 Supplement 1, 2015: Taking Stock of Reproductive Health in Humanitarian Settings: 2012-2014 Inter-agency Working Group on Reproductive Health in Crises' Global Review. The full contents of the supplement are available online at http://www.conflictandhealth.com/supplements/9/S1. Funding for this supplement was provided by the MacArthur Foundation.
